# Mood Disorder Questionnaire Positivity in Systemic Lupus Erythematosus and Other Chronic Diseases including Screen Bipolar Disorders or Rhythm and Energy Dysregulation Syndromes (DYMERS)

**DOI:** 10.2174/0117450179303653240705051227

**Published:** 2024-08-08

**Authors:** Diego Primavera, Michele Fornaro, Giuseppe Carrà, Ferdinando Romano, Cesar Ivan Aviles Gonzales, Antonio Preti, Federica Sancassiani, Giulia Cossu, Antonio Egidio Nardi, Alessandra Scano, Germano Orrù, Elisabetta Chessa, Alberto Floris, Matteo Piga, Alberto Cauli, Mauro Giovanni Carta

**Affiliations:** 1 Department of Medical Sciences and Public Health, University of Cagliari, Italy Section of Psychiatry, Cagliari, Italy; 2 Department of Neuroscience, Reproductive Science and Odontostomatology Federico II University of Naples, Naples, Italy; 3 Department of Medicine and Surgery, University of Milano-Bicocca, Via Cadore 48, Monza 20900, Italy; 4 Division of Psychiatry, University College London, 149 Tottenham Court Road, London, W1T 7NF, UK; 5 Department of Public Health and Infectious Diseases, Sapienza University of Rome, Rome 00185, Italy; 6 Nursing Program, Faculty of Health Sciences, Universidad Popular del Cesar, Valledupar, Colombia; 7 Laboratory Panic and Respiration, Institute of Psychiatry (Ipub), Federal University of Rio De Janeiro (Ufrj), Rio De Janeiro 22725, Brazil; 8 Department of Neuroscience, University of Turin, Turin 10125, Italy; 9 Institute of Psychiatry, Federal University of Rio de Janeiro, Brasil; 10 Department of Surgical Sciences, University of Cagliari, Cagliari, Italy; 11 Department of Medical Sciences and Public Health, University of Cagliari, Monserrato 09042, Italy; 12 Rheumatology Unit, Azienda Ospedaliero Universitaria di Cagliari, Monserrato 09042, Italy

**Keywords:** SLE, Bipolar disorder, Rhythm dysregulation, New syndrome, MDQ, DYMERS

## Abstract

**Introduction:**

This study explores the issue of paper-and-pencil screening tests for bipolar disorder, often leading to false positives. It discusses hypotheses that connect MDQ positivity with sleep disorders, a decline in health-related quality of life, and the impact of the COVID-19 pandemic on mood disorders. The study proposes that MDQ identifies a “Dysregulation of Mood, Energy, and Social Rhythms Syndrome” (DYMERS), indicating a stress-related condition. It aims to investigate the association between MDQ positivity and systemic lupus erythematosus (SLE) in comparison to other chronic disorders.

**Methods:**

This case-control study, conducted from April 2019 to February 2020, investigated MDQ positivity in patients with SLE. Ethical approvals were obtained, and statistical analysis was used for data assessment.

**Results:**

This is a case-controlled study where MDQ positivity was significantly higher in systemic lupus erythematosus cases than controls. The analysis compared gender, age, and the presence of depressive episodes between MDQ-positive and MDQ-negative cases, revealing some differences but no significant variations. Interestingly, no association with high prednisone or biologics use was observed. The frequency of MDQ positivity in systemic lupus erythematosus was compared to other chronic pathologies, revealing varying associations with each condition.

**Conclusion:**

This study reveals a high rate of (MDQ) positivity in systemic lupus erythematosus (SLE), associated with the risk of bipolar disorder in SLE. Notable discrepancies in MDQ positivity risk factors between SLE and bipolar disorder are observed. The study emphasizes the ability of MDQ to identify a distinct syndrome characterized by rhythm dysregulation, posing a risk for bipolar disorder and other disorders.

## 1 INTRODUCTION

Inconclusive evidence exists about the prevalence rates of bipolar disorder across different literature sources, with both evidence in support and the discharge of over- and under-misdiagnosis, with a special reference to sub-threshold bipolarity among people endorsing a major depressive episode [1].

Validated rating tools for detection against validated semi-structured interviews (regarded as the gold standard for the diagnosis of BD along with a comprehensive clinical evaluation) may yield different psychometric performances across different clinical populations, settings, and cultural/geographical adaptations, sparking a vivid debate about their clinical and epidemiological significance, especially in case of positive screening at a paper-and-pencil screening test, warranting additional research on the matter [2].

For example, some tools may inflate the rates of false positive diagnoses of BD [3], particularly in the occurrence of BD-related conditions sharing marked impulsivity and affective instability [4-6].

Many individuals who test positive find themselves in the realm of alternative psychiatric diagnoses, such as anxiety disorders, attention-deficit/hyperactivity disorder (ADHD), post-traumatic stress disorder (PTSD), and various personality disorders [7, 8]. Up to one-quarter of these patients may wait an average of 10-15 years for a formal BD diagnosis, possibly with burdensome consequences [9].

The ongoing debate about the validity of the screening tools for BD revolves around the complexities inherent in identifying BD. Structured interviews, considered the gold standard in psychiatric assessment, present a dilemmanamely, the substantial occurrence of false positives.

These false positives, when uncovered, cast individuals into the shadowy realm of alternative psychiatric conditions. Anxiety disorders, ADHD, PTSD, and personality disorders often become conceivable diagnoses. This debate underscores the intricate and multifaceted nature of BD diagnosis. It beckons researchers and clinicians to navigate the labyrinthine path of diagnostic criteria and screening tools, striving for greater accuracy and precision in identifying this complex mood disorder.

On the opposing front, proponents of the neo-Kraepelinian approach, particularly influential within the European psychiatric community, assert a strikingly different perspective. Their argument hinges on the premise that the prevailing diagnostic criteria, such as DSM-5 are unduly rigid and insufficient in encompassing the expansive spectrum of BDs [10, 11].

This debate unveils a critical point of contention, highlighting the inherent tensions surrounding the characterization of bipolar disorders. The neo-Kraepelinian proponents challenge the established norms, advocating for a more flexible and encompassing approach to diagnosis. This approach not only acknowledges the inherent complexity of BDs but also seeks to redefine the boundaries that currently circumscribe our understanding of these conditions.

People identified as “false positives” through screening tests, such as the MDQ screener, exhibit a constellation of clinical features requiring meticulous examination [12-14]. These individuals often bear a striking resemblance to cases diagnosed with full-blown bipolar disorders, both in terms of symptomatology and in the familial patterns that emerge. Furthermore, the risk factors that underpin their condition are uncannily similar to those observed in established cases of BD [2].

The intrigue deepens as we consider the temporal dimension of these conditions. The disorders identified among these presumed false positives often coexist with bipolar disorders, forming a web of comorbidity that intertwines their fates. Intriguingly, these conditions may precede the emergence of bipolar disorders by several years. This temporal relationship invites contemplation of a potential causal interplay, raising questions about whether these seemingly distinct conditions share etiological underpinnings or represent interconnected facets of a broader, multifaceted spectrum.

In essence, this dispute embodies the fundamental tension between two divergent paradigms in psychiatric diagnosis. It serves as a reminder that the complexities inherent in understanding and characterizing mental health conditions, particularly those as intricate as bipolar disorders, continue to challenge our diagnostic frameworks and inspire ongoing reevaluation and refinement.

Recent advancements have ushered in a new era of understanding this intricate matter, offering a multifaceted perspective on the subject:

### 1.1 Sleep Disorders and MDQ Positivity

A significant revelation in recent research is the association between MDQ positivity and sleep disorders, irrespective of whether comorbid mood disorders are present. This groundbreaking insight unveiled [15] under-scores the intricate interplay between sleep disturbances and MDQ screening outcomes. It suggests that sleep disorders may be an independent factor contributing to MDQ positivity, thus unraveling a novel dimension of this complex relationship.

### 1.2 MDQ Positivity and Health-related Quality of Life

Another compelling discovery is the profound impact of a positive MDQ score on an individual's health-related quality of life. This phenomenon, explored in several studies [16, 17]and the forthcoming research, reveals that the ramifications of MDQ positivity extend far beyond diagnostic labels. Notably, this effect transcends comorbid mood disorders and other psychiatric conditions, indicating that even in the absence of these traditional qualifiers, MDQ positivity leaves an indelible mark. This revelation gains additional intrigue when extended to older adults, a demographic where the likelihood of developing a mood disorder in the future is ostensibly low. It prompts reflection on the lasting impact of MDQ positivity on the well-being and life satisfaction of individuals, regardless of age [16].

### 1.3 Dysregulated Social and Behavioral Rhythms During COVID-19

The global upheaval caused by the COVID-19 pandemic, with its widespread lockdowns and societal disruptions, has shone a spotlight on the pivotal role played by dysregulated social and behavioral rhythms, particularly sleep, in the exacerbation of critical episodes within the realm of mood disorders. As illuminated in a previous study [18], this finding underscores the intricate interplay between external environmental factors and the manifestation of mood disorders. The profound implications of this revelation resonate in a world grappling with the consequences of a pandemic, hinting at the broader implications for public health and well-being.

### 1.4 Emotional Hyperreactivity to Stress and Trauma

Recent investigations have yielded a wealth of evidence about emotional hyperreactivity in response to stress and trauma. This burgeoning body of research, including the work of Giotakos O. *et al*. in 2020 and Herringa in 2017, unravels the complex dynamics that underlie emotional responses in individuals facing challenging life circumstances. It sheds light on the heightened reactivity observed in the face of stressors and traumatic experiences, challenging our understanding of emotional resilience and vulnerability [19, 20].

In summation, these recent findings represent a paradigm shift in our comprehension of the intricacies surrounding MDQ positivity, its impact on individuals, and its broader implications in the context of evolving societal and environmental challenges. They invite further exploration, providing a richer tapestry of insights that shape our approach to understanding and addressing mood disorders and related conditions.

As a result, it has been postulated that MDQ positivity identifies a condition termed Dysregulation of Mood, Energy, and Social Rhythms Syndrome (DYMERS), a state of severe stress that, depending on individual predis-position, can serve as the initial, nonspecific manifestation of various psychiatric disorders [21]. In the case of bipolar disorder, individuals with a predilection for exploration and novelty-seeking tendencies, combined with a genetic predisposition that need not be pathological [22, 23], could eventually manifest explicit bipolar disorder under the influence of substantial stress, with DYMERS acting as the initial, unspecific presentation [24].

One recent study aimed to assess the accuracy and reliability of the Mood Disorder Questionnaire (MDQ) and a genetic variant (RS1006737 of CACNA1C) as screening tools for bipolar disorder (BD) in older adults. The findings indicated that these tests were not dependable when used in isolation, highlighting the need for a combined approach. These results suggested a potential link between hyperactivity-related conditions and BD, especially in individuals with a genetic predisposition [25]. Another study explored whether individuals with a positive score on the Mood Disorder Questionnaire (MDQ) but without mood disorders exhibited lower Health-Related Quality of Life (HRQoL) compared to a control group. The findings pointed to the existence of a “Dysregulation of Mood, Energy, and Social Rhythms Syndrome” (DYMERS), characterized by significant distress unrelated to other disorders. This may contribute to the development of associated conditions, including bipolar disorder [21].

The primary objective of this study was to investigate the relationship between MDQ positivity and systemic lupus erythematosus (SLE), a chronic disorder known to impact psychosocial functioning and social rhythms.

Systemic Lupus Erythematosus (SLE), commonly referred to as lupus, is a complex and multifaceted autoimmune disease. This disorder primarily affects women, with a female-to-male ratio of about 9:1, and it often has its onset during the reproductive years. However, it can affect individuals of all ages. SLE is characterized by a wide range of clinical manifestations, which can involve various organ systems, and it is known for its unpredictable and relapsing-remitting course [26].

The exact cause of SLE remains elusive, but it is believed to result from a combination of genetic, environmental, and hormonal factors. There is strong evidence of a genetic predisposition, with several susceptibility genes identified, including HLA-DR, PTPN22, and STAT4. Environmental factors, such as exposure to ultraviolet light, certain infections, and hormonal fluctuations, can trigger the disease in genetically susceptible individuals [27].

SLE is an autoimmune disease characterized by the aberrant activation of innate and adaptive immune responses. The production of autoantibodies against a wide array of self-antigens leads to immune complexes deposit in various tissues and organs, leading to inflammation and tissue damage [28]. Brain-reactive autoantibodies may contribute to the pathogenesis of neuropsychiatric SLE [29, 30].

SLE is known for its protean clinical manifestations, which can affect virtually any organ system. Common symptoms include arthralgia, fever, skin rashes (*e.g*., malar rash), and fatigue. More severe manifestations can involve the kidneys (lupus nephritis), central nervous system (neuropsychiatric lupus), joints (arthritis), serosae (pleuritis, pericarditis), and hematological abnormalities (anemia, leukopenia, thrombocytopenia).

The diagnosis of SLE is clinical, and its classification is based on criteria established by the American College of Rheumatology (ACR) and the European Alliance of Associations for Rheumatology (EULAR). These criteria encompass clinical and laboratory features, including autoantibodies [31].

The management of SLE aims to control disease activity, prevent organ damage, and improve patients' quality of life, with a treatment plan including non-steroidal anti-inflammatory drugs (NSAIDs) for mild symptoms, corticosteroids, and immunomodulating/ immunosuppressive agents (*e.g*., hydroxychloroquine, mycophenolate mofetil, belimumab) for more severe disease. Individualized treatment plans are crucial, considering the heterogeneous nature of the disease and its variable course [32].

While the prognosis of SLE has significantly improved, severe organ involvement, such as lupus nephritis or neuropsychiatric manifestations, can lead to significant morbidity and mortality. Regular monitoring and close follow-up are essential for optimizing patient outcomes [33].

We aim to compare this association with the link between MDQ positivity and other chronic disorders, as reported in prior analogous studies. By examining the distribution of MDQ positivity, we will explore its alignment with the prevalence of bipolar disorder within various chronic diseases.

## 2 METHODS

### 2.1 Design

The study adopted a case-control design.

### 2.2 Study Sample

 Patients were consecutively recruited for the study from April 2019 to February 2020 at the Rheumatology Unit of the University Hospital of Cagliari. The inclusion criteria were≥18 years, being capable of giving consent, and diagnosis of SLE according to ACR/EULAR 2019 criteria. Disease activity was assessed by the Systemic Lupus Erythematosus Disease Activity Index 2000 (SLEDAI-2K) [35] and Physician Global Assessment (PGA) [35]. The exclusion criteria for our study were the inability to adequately sign the informed consent and a low educational level that precluded the ability to read and write. The control group was chosen after randomization by blocks from the database of a community survey [36]. A matched control cell was created for each case with all the people without SLE of the same age and sex of the case drawn from the databank. Four controls were randomly selected in each cell. Once a healthy subject was included in a block, he was excluded from the eligibility for the remaining blocks. Given the experience from previously published studies on patients with chronic conditions testing positive on the MDQ, the sample size was calculated based on these studies [37-41]

### 2.3 Tools and Assessment

 The Italian version of the Mood Disorder Questionnaire (MDQ) was used to assess lifetime hypomanic episodes or episodes of augmentation of energy [42] 28. The “Patient Health Questionnaire” [43] 29 Italian [44] 30 version with 9 items (PHQ-9) was used for screening the depressive episodes. The tool investigates all the nine core DSM criteria for the depressive episode. To screen mild/moderate MDD, a cut-off of the total score of ≥ 7 is considered quite accurate [44]. *An Ad-hoc* form was adopted to collect socio-demographic and anamnestic data.

### 2.4 Ethical Aspects

 The study has the approbation of the Ethical Committee of the Independent Ethics Committee of the University Hospital of Cagliari [number PG/2019/4522). The Italian community survey, for which data database was used for the choice of the controls, had the approbation of the ethical committee of the Italian National Health Institute (Rome) [ISTISAN 200^8^]. The protocol predicted the possibility of use of the databank for control for conducting case-control studies. The survey was conducted according to the 1964 Helsinki Declaration and amendments. All the participants of the study sample signed a written informed consent after a full description of the aims, the procedures, the data protection, and the possibility to terminate the study at any time.

### 2.5 Statistical Analyses

 Data were anonymously collected by an ID number for each subject, and data entry was made in a dedicated database. The one-way ANOVA statistic was adopted to measure differences in numerical data, and the Chi-square test was used to compare nominal data.

## 3 RESULTS

The study sample comprised a total of 32 participants, and 128 controls were drawn from a sample of the Italian general population.

The differences between the study sample and the controls are as follows:

### 3.1 Sex

Both the study and the controls exhibited a percentage of 90.62% female participants, indicating a perfect match between the two groups regarding gender.

### 3.2 Age

Both the study and the controls had a mean age of 440 years with a standard deviation of ±13.64, indicating a perfect match also regarding age.

### 3.3 MDQ+ (Presence of Bipolar Disorder)

In the study group, 8 (25%) of the participants tested positive for MDQ+ (indicative of bipolar disorder), whereas in the control group, only 8 (6.25%) of the participants had the same result. This difference was statistically significant using the Chi-square test with Yates correction (χ^2^=825, 1df, p=005), with an odds ratio (OR) of 50 and a 95% confidence interval (CI95%) ranging from 1.71 to 14.63.

Table **1** illustrates the characteristics of the case and control samples; age and gender are perfectly balanced due to the block randomization technique. The case group presents a higher frequency of MDQ positives (25% *versus* 6.25% OR=50 CI 95% 1.71-14.63).

Table **2** provides a comparative analysis between individuals testing positive (MDQ+) and negative (MDQ-) on the MDQ test across various factors, with sample sizes denoted as N=8 for MDQ+ and N=22 for MDQ. Statistical tests were conducted to assess the significance of the differences observed. The frequency of gender and age did not present statistical significance differences, although a tendency towards a younger age emerged among the MDQ-positive cases (38.25 ±104 in MDQ+ *versus* 45.91±14.84; ANOVA 1 way, df 1, 30.3, F=1.830, P=0.186). The presence of depressive episodes identified by a PHQ9 score>7 did not differ in the two groups (25% in MDQ+ *vs* 37.5%, OR=0.55 CI95% 09-3.65). Taking high amounts of prednisone and/or biologics was not associated with MDQ positivity. All cases with MDQ positivity took combinations of antimalarials and immunosuppressants (100% *vs* 54.15% in controls, Fisher Exact Test, p=002, OR=inf CI95% Not calculable).

Table **3** compares the frequency of MDQ positives in Systemic Lupus Erythematosus and other chronic pathologies related to the same public clinical center (therefore from the same catchment area) that produced the cases of this study (University Hospital of Cagliari). The frequency of MDQ positivity in Systemic Lupus Erythematosus was higher than in Celiac Disease (25% *vs* 6.7%, OR=4.67, CI95% 1.28-16.98) and in Multiple Sclerosis (25% *vs* 9.9%, OR=32, CI95% 1.20-7.60), no differences were found with Wilson's disease and carotid atherosclerosis, the frequency was instead lower than with fibromyalgia syndrome (25% *vs* 59.4%, OR=0.22, CI95% 08-0.64).

Table **4** illustrates the frequency of MDQ+ found in the cases compared with that found with the same system in a sample of the general Italian population. All the chronic pathologies considered had a higher frequency than the general population except celiac disease (6.7% *vs* 3%, OR=2.28, CI95% 0.81-6.42). The closest association was with Fibromyalgia (OR=48.92, CI 05% 23.65-937) following Wilson's disease (OR=20.56, CI 95%, 0.16-48.70); these conditions preceded Systemic Lupus Erythematosus (OR= 10.66, 95% CI 4.68-24.30) showing higher frequencies of carotid atherosclerosis (OR=4.80, 95% CI 1.99-110) and Multiple Sclerosis (OR=3.53, 95% CI 2.14-5.84).

## 4 DISCUSSION

The study found a high frequency of MDQ positivity in systemic lupus erythematosus compared to a matched community sample. The frequency in SLE was like that found in case-control studies conducted on chronic disabling conditions, such as Wilson's disease and carotid atherosclerosis, higher than the frequency found in celiac disease and multiple sclerosis and lower only than the frequency of MDQ positivity found in fibromyalgia. All these disorders examined had a higher frequency of MDQ+ than those found in the general Italian population, except for celiac disease. Although this comparison was conducted without standardization for the major confounding variables, the case-control study (with a balanced sample) still found that there was no statistically significant difference between the frequency of MDQ positivity in the sample of Celiac Disease under examination and the sample of standardized control of the general population, while on the contrary, a greater frequency in Celiac Disease of Bipolar Disorder was found. The high risk for bipolar disorder in celiac disease is, on the other hand, confirmed by other research [45].

A certain discrepancy between the distribution of MDQ positivity and that of full-blown bipolar disorder (Diagnosis of BD I or BD II according to DSM-IV) is evident if you consider the very high frequency of MDQ+ found in fibromyalgia. Indeed, FMs have also been found to be associated with bipolar disorder. Still, in a case-control study, the frequency was lower than, for example, of Wilson's disease (21.12% in Fibromyalgia Syndrome against 30,4% in Wilson's disease) [46]. On the contrary, if we consider the frequency of MDQ positivity, the fibromyalgia syndrome reaches 59.4% against only 39.1% in Wilson’s disease [47]. From this point of view, one of the most peculiar clinical characteristics of fibromyalgia is the regulation of circadian rhythms and behavioral rhythms, particularly of sleep. As previously observed, MDQ positivity is shown to be closely associated with sleep dysregulation [46].

The imperfect overlap of risk factors between bipolar disorder and MDQ positivity is also evident if we consider the emerging risk factors for MDQ in our sample. MDQ+ was not associated with depressive episodes or the intake of high doses of prednisone, two elements strongly associated with bipolar disorder [47], according to Brown (2009) [48], Gable and Depry (2015). It is also of interest how positivity to the MDQ was, on the contrary, closely associated with the intake of antimalarial and immuno-suppressive drugs for which a direct induction of bipolar disorders is not known as a side effect, even if current research highlights how immunosuppressive drugs can directly induce an alteration of circadian biorhythms (in close relation with behavioural rhythms) such as sleep [49].

Our research confirms that the risk of MDQ positivity occurs in conditions in which even the risk of bipolar disorder is high (as in almost all the chronic conditions analysed). Indeed, despite the recorded high frequency of “false positives”, the MDQ screener identifies a large portion of individuals whose symptoms reach the threshold of diagnosis for bipolar disorder [8], according to Zimmerman and Galione, 2011 [1]. However, there are some discrepancies in the distribution and risk factors between MDQ+ and Bipolar Disorder that seem to confirm, on the one hand, that the MDQ captures only one component of bipolar disorder, as recently demonstrated [25]. On the other hand, positivity to the MDQ reveals a specific syndrome of hyperactivation and dysregulation of rhythms, which is a risk condition for bipolar disorder but can also be a vulnerable condition for other disorders [21]. This would also explain the high frequency of comorbidities but also the impairment of the quality of life, which is often associated with positivity to the MDQ even without comorbidities and in the elderly in whom future co-morbidity is difficult to hypothesize if, at the time of the evaluation, there was no lifetime comorbidity.

Along with these observations, our study indicates a high risk of MDQ positivity in patients with Lupus. This is also in relation (but probably not only, as previously discussed) to an evident high risk for bipolar disorder.

This result found some confirmations in the literature [50] but, if confirmed, would also be of extreme interest due to the implication in the neuro-lupus [51] and for the choice of the therapies conducted for the rheumatological disease considering that some of them have bipolar disorders as a side effect [52]. The limitations of our study lie in the case-control design and the small sample size. However, the importance of the topics suggests the future conduct of prospective studies on large samples.

## CONCLUSION

In conclusion, this study has highlighted the prevalence of MDQ positivity within SLE, offering valuable insights and raising several compelling observations. The salient findings and the broader implications of this research are summarized as follows:

## MDQ POSITIVITY IN SLE

The study unveiled a significant prevalence of MDQ positivity in individuals with SLE compared to a matched community sample. This frequency was notably akin to other chronic debilitating conditions, including Wilson's disease and carotid atherosclerosis. Notably, the frequency of MDQ positivity in SLE surpassed that found in conditions, such as celiac disease and multiple sclerosis, albeit remaining slightly lower than the prevalence observed in fibromyalgia. The common thread among these conditions was a higher MDQ+ frequency when compared to the general Italian population, except for celiac disease. This observation not only underscores the significance of MDQ positivity within these chronic conditions but also highlights its potential utility as an indicator of underlying disorders.

## MDQ POSITIVITY AND VARIATIONS ACROSS CONDITIONS

When further dissecting the relationship between MDQ positivity and full-blown bipolar disorder, discrepancies emerged. Notably, the frequency of MDQ+ cases in fibromyalgia was markedly high despite fibromyalgia having a lower prevalence of bipolar disorder compared to Wilson's disease. This raises questions about the complex interplay between MDQ positivity and bipolar disorder across different chronic conditions.

## EXPLORING RISK FACTORS FOR MDQ POSITIVITY

The study found that MDQ positivity was not strongly associated with depressive episodes or the use of high doses of prednisone, both of which are traditionally linked to bipolar disorder. Conversely, MDQ positivity exhibited a notable correlation with the use of antimalarial and immunosuppressive drugs, which are not commonly known to induce bipolar disorder. Current research suggests these drugs may impact circadian and behavioral rhythms, particularly sleep, adding depth to our understanding of the factors influencing MDQ positivity.

## MDQ POSITIVITY AND BIPOLAR DISORDER RISK

Despite the presence of “false positives,” the MDQ screener effectively identified a substantial number of individuals whose symptoms aligned closely with the threshold for bipolar disorder diagnosis. While disparities exist between MDQ+ and full-blown bipolar disorder, the MDQ screener appears to capture a distinct syndrome characterized by hyperactivation and rhythm dysregulation. This syndrome not only poses a risk for bipolar disorder but also emerges as a vulnerable condition for other disorders, potentially explaining the high comorbidity rates and quality of life impacts, even in individuals without comorbidities, especially among the elderly.

## IMPLICATIONS FOR LUPUS PATIENTS

Notably, the study highlights a considerable risk of MDQ positivity in patients with SLE, suggesting a potential connection to an increased risk of bipolar disorder. These findings align with existing literature but also bring to the forefront the implications for neuro-lupus and the choice of therapies for rheumatological diseases. This connection, if substantiated, could have far-reaching consequences for the treatment and care of individuals with lupus.

## LIMITATIONS AND FUTURE DIRECTIONS

While this study offers valuable insights, it is essential to acknowledge its limitations, primarily due to its case-control and relatively small sample size. The complexities of the subject and the need for a more comprehensive understanding underscore the importance of future prospective studies involving larger and more diverse cohorts.

This research provides a valuable stepping stone in our comprehension of MDQ positivity, its associations with chronic conditions like lupus, and its associations with bipolar disorder. It underscores the multifaceted nature of MDQ positivity, encouraging further exploration into the complexities of this phenomenon in psychiatry and beyond.

## AUTHORS’ CONTRIBUTIONS

It is hereby acknowledged that all authors have accepted responsibility for the manuscript's content and consented to its submission. They have meticulously reviewed all results and unanimously approved the final version of the manuscript.

## Figures and Tables

**Table 1:** Study and control samples (matched by sex and age).

**Study Sample**		Controls*	**Differences**
-	**N (%)**	**N (%)**	-
**Female**	29 (90.62)	116 (90.62)	Perfect matched
**Age**	440±13.64	440±13.64	Perfect matched
**MDQ+**	8 (25%)	8 (6.25%)	Chi-square with Yates correction=825, 1df, p=005 OR=50 CI95% 1.71-14.63
**Total**	32	128	-

Note: *From a community sample of the Italian general population.

**Table 2:** Characteristics of MDQ+ *versus* MDQ- in SLE.

**Factor**	**MDQ+** **(N=8)**	**MDQ-** **(N=22)**	**Statistics**
**Female**	7 (87.5%)	22(91.6%)	Chi-square with Yates correction=001, 1df, p=0.999 OR=0.63 CI95% 05-8.12
**Age** **MDQ+/MDQ-**	38.25±104	45,91±14.84	ANOVA 1 way, df 1, 30,31 F=1.830 P=0.186
**PHQ9+** **MDQ+/MDQ-**	2 (25%)	9 (37.5%)	Chi-square with Yates correction=046, 1df, p=0.830 OR=0.55 CI95% 09-3.65
**Prednisone ≥5mg**	3 (37.5%)	17 (70.8%)	Fisher Exact Test, p=0.116 OR=0.25 CI95% 05-1.32
**Antimalarials + Immunosuppressants**	8(100%)	13 (54.15%)	Fisher Exact Test, p=002 OR=inf CI95% NC
**Biologicals**	4 (50%)	20(83.3%)	Fisher Exact Test, p=0.116 OR=0.20 CI95% 04-1.15

**Table 3:** MDQ+ in chronic diseases (comparison with SLE).

**Disease**	**MDQ Frequency**	Comparison with Systemic Lupus Erythematosus Chi-square 1 df (with Yates Correction if needed), P	OR (CI 95%)
Multiple Sclerosis (Italy 2011-2013) [38]	20 [9.9%] (N=201)	Χ^2^=4.576; p=032	32 (1.20-7.60)
Wilson’ Disease (Italy 2010) [39]	9 [39.1%] (N=23)	Χ^2^=6.277; p=0.411	0.52 (0.16-1.65)
Carotidal atherosclerosis (Italy 2013-2014) [40]	6 [13%] (N=46)	Χ^2^=0.292; p=0.176	2.22 (0.69-7.18)
Celiac Disease (Italy 2014-2015) [41]	4 [6.7%] (N=60)	Χ^2^=4.674; p=031	4.67 (1.28-16.98)
Fibromyalgia (Italy 2004-2005) [42]	22 [59.4%] (N=37)	Χ^2^=6.949; p=008	0.22 (08-0.64)
Systemic Lupus Erythematosus	8 [25%] (N=32)	Pivot	-

Note: *Different cut-off (4 positive answers plus impairment).

**Table 4:** MDQ positives in chronic diseases comparison with Italian unbalanced community sample.

**Disease**	**MDQ Frequency**	**Comparison with an Unbalanced Community Sample** **Chi-square 1 df** (with Yates Correction if needed), P	OR (CI 95%)
Multiple Sclerosis (Italy 2011-2013) [38]	20 (201) [9.9%]	Χ^2^=27.524; p<001	3.53 (2.14-5.84)
Wilson’ Disease (Italy 2010) [39]	9 (23) [39.1%]	Χ^2^=82.958; p<001	20.56 (0.16-48.70)
Carotidal atherosclerosis (Italy 2013-2014) [40]	6 (46) [13%]	Χ^2^=11.758; p=001	4.80 (1.99-110)
Celiac Disease (Italy 2014-2015) [41]	4 (60) [6.7%]	Χ^2^=1.526; p=0.216	2.28 (0.81-6.42)
Fibromyalgia (Italy 2004-2005) [42]	22(37) [59,4%]	Χ^2^=316.463; p<001	48.92 (23.65-937)
Systemic Lupus Erythematosus	8 (25%) [N=32]	Χ^2^=4296; p<001	10.66 (4.68-24.30)
Unbalanced community sample	103 (3%) [3398]	Pivot	-

## Data Availability

The data and supportive information are available within the article.

## LIST OF ABBREVIATIONS

ADHD: Attention-deficit/hyperactivity Disorder
PTSD: Post-traumatic Stress Disorder
BD: Bipolar Disorder
MDQ: Mood Disorder Questionnaire
SLE: Systemic Lupus Erythematosus

## STANDARDS OF REPORTING

STROBE guidelines were followed.
