# Corrigendum to: Mood Disorder Questionnaire Positivity in Systemic Lupus Erythematosus and Other Chronic Diseases

**DOI:** 10.2174/0117450179303653240702409021

**Published:** 2024-09-04

**Authors:** Diego Primavera, Michele Fornaro, Giuseppe Carrà, Ferdinando Romano, Cesar Ivan Aviles Gonzales, Antonio Preti, Federica Sancassiani, Giulia Cossu, Antonio Egidio Nardi, Alessandra Scano, Germano Orrù, Elisabetta Chessa, Alberto Floris, Matteo Piga, Alberto Cauli, Mauro Giovanni Carta

**Affiliations:** 1 Department of Medical Sciences and Public Health, University of Cagliari, Italy Section of Psychiatry, Ca-gliari, Italy; 2 Department of Neuroscience, Reproductive Science and Odontostomatology Federico II University of Na-ples, Naples, Italy; 3 Department of Medicine and Surgery, University of Milano-Bicocca, Via Cadore 48, Monza 20900, Italy; 4 Division of Psychiatry, University College London, 149 Tottenham Court Road, London, W1T 7NF, UK; 5 Department of Public Health and Infectious Diseases, Sapienza University of Rome, Rome 00185, Italy; 6 Nursing Program, Faculty of Health Sciences, Universidad Popular del Cesar, Valledupar, Colombia; 7 Laboratory Panic and Respiration, Institute of Psychiatry (Ipub), Federal University of Rio De Janeiro (Ufrj), Rio De Janeiro 22725, Brazil; 8 Department of Neuroscience, University of Turin, Turin 10125, Italy; 9 Institute of Psychiatry, Federal University of Rio de Janeiro, Brazil; 10 Department of Surgical Sciences, University of Cagliari, Cagliari, Italy; 11 Department of Medical Sciences and Public Health, University of Cagliari, Monserrato 09042, Italy; 12 Rheumatology Unit, Azienda Ospedaliero Universitaria di Cagliari, Monserrato 09042, Italy

In the online version of the article, a change was made in the title of an article titled “**Mood Disorder Questionnaire Positivity in Systemic Lupus Erythematosus and Other Chronic Diseases including Screen Bipolar Disorders or Rhythm and Energy Dysregulation Syndromes (DYMERS)**” which has been updated in “**Clinical Practice and Epidemiology in Mental Health**,” 2024; 20: e17450179303653 [1].

The original article can be found online at: https://clinical-practice-and-epidemiology-in-mental-health.com/VOLUME/20/ELOCATOR/e17450179303653/FULLTEXT/

## Original:

Mood Disorder Questionnaire Positivity in Systemic Lupus Erythematosus and Other Chronic Diseases including Screen Bipolar Disorders or Rhythm and Energy Dysregulation Syndromes (DYMERS)

## Corrected:

Mood Disorder Questionnaire Positivity in Systemic Lupus Erythematosus and Other Chronic Diseases

## References

[1] Primavera D., Fornaro M., Carrà G., Romano F., Ivan C., Gonzales A., Preti A., Sancassiani F., Cossu G., Egidio A.N., Scano A., Orrù G., Chessa E., Floris A., Piga M., Cauli A., Carta M.G. (2024). Mood Disorder Questionnaire Positivity in Systemic Lupus Erythematosus and Other Chronic Diseases.. Clin. Pract. Epidemiol. Ment. Health.

